# Adipose Stem Cells: From Bench to Bedside

**DOI:** 10.1155/2016/6484038

**Published:** 2016-03-21

**Authors:** Giuseppe A. Ferraro, Hiroshi Mizuno, Norbert Pallua

**Affiliations:** ^1^Multidisciplinary Department of Medical-Surgical and Dental Specialties, Second University of Naples, 80138 Naples, Italy; ^2^Department of Plastic and Reconstructive Surgery, Juntendo University School of Medicine, Tokyo 1138421, Japan; ^3^Department of Plastic Surgery, Reconstructive and Hand Surgery, Burn Center, RWTH University Hospital Aachen, 52062 Aachen, Germany

Stem cell biology plays an important role in promoting cell-based treatment. Adult mesenchymal stem cells (MSCs) are derived from various tissues including bone marrow [1], adipose tissue [2], dental pulp [3], and Wharton jelly [4]. When compared to bone marrow mesenchymal stem cells (BM-MSCs), adipose tissue represents an ideal source for multipotent progenitors in adults [5]. Adipose stem cells (ASCs) share many characteristics with BM-MSCs, including extensive proliferation and the ability to undergo multilineage differentiation [6] (like bone marrow MSCs, they can differentiate* in vitro* into adipogenic, osteogenic, chondrogenic, and myogenic cells when cultured in specific lineage-inducing culture media and into endothelial cells), and they display a noticeable plasticity both* in vitro* and* in vivo*. Moreover, the high abundance of adipose tissue within the body, its high surgical accessibility, and the demonstrated multipotency of ASCs show adipose tissue as a promising candidate for MSCs harvest and increase the interest in its use in tissue repair, regenerative medicine, and degenerative disease management [7].

ASCs have been studied widely since stem cell investigations emerged in 2001 [8]. Since then, knowledge of their characterization, immunological characteristics, and potential of multilineage differentiation has increased considerably [9–18]. Many international medical conferences have emphasized the importance of ASCs and the International Federation of Adipose Therapeutics and Science (IFATS) has been extremely active in promoting the study and discussion of ASCs [19].

Many surgical strategies for tissue loss replacement initially focused on the historical maxim “replace tissue with like-tissue” procedure. In more recent years, several allogenic and alloplastic materials have been developed and used for tissue repair [20–22]. Current research aims to reduce concerns such as foreign-body reactions, rapid degradation, and risk of immunogenicity.

The development of regenerative medicine strategies requires an appropriate cell source and scaffold, “smart” biomaterials (novel “intelligent” biomaterials with appropriate physical properties able to support* in vivo* the commitment of adipose stem cells), and a suitable microenvironment to provide the cues and signals for cell growth and tissue formation. Biomaterials are able to direct and organize the cellular events involved in the regenerative process* in situ* [23, 24]. ASCs are undifferentiated cells with the ability to self-renew and differentiate into different types of specialized cells with a regenerative potential even if not combined with biomaterials. The proliferation and differentiation of adipose stem cells can be regulated biochemically, as well as through the physical properties of microenvironments, such as the topography of the scaffolds, the “stiffness,” and mechanical forces.

The potential of adipose stem cell therapies and regenerative medicine is effective and challenging, offering the possibility of tissue repair and replacement in tissue defects related to congenital diseases, trauma, and cancer [25].

This special issue has examined the importance of “adipose stem cells” focusing on the basic biology and potential role of ASCs in the treatment and regeneration of cells, tissues, and organs.
